# Inhibition of GLUD1 mediated by LASP1 and SYVN1 contributes to hepatitis B virus X protein-induced hepatocarcinogenesis

**DOI:** 10.1093/jmcb/mjae014

**Published:** 2024-04-08

**Authors:** Hong-Juan You, Qi Li, Li-Hong Ma, Xing Wang, Huan-Yang Zhang, Yu-Xin Wang, En-Si Bao, Yu-Jie Zhong, De-Long Kong, Xiang-Ye Liu, Fan-Yun Kong, Kui-Yang Zheng, Ren-Xian Tang

**Affiliations:** Jiangsu Key Laboratory of Immunity and Metabolism, Department of Pathogenic Biology and Immunology, Xuzhou Medical University, Xuzhou 221004, China; Jiangsu Key Laboratory of Immunity and Metabolism, Department of Pathogenic Biology and Immunology, Xuzhou Medical University, Xuzhou 221004, China; Laboratory Department, The People's Hospital of Funing, Yancheng 224400, China; Jiangsu Key Laboratory of Immunity and Metabolism, Department of Pathogenic Biology and Immunology, Xuzhou Medical University, Xuzhou 221004, China; Jiangsu Key Laboratory of Immunity and Metabolism, Department of Pathogenic Biology and Immunology, Xuzhou Medical University, Xuzhou 221004, China; Jiangsu Key Laboratory of Immunity and Metabolism, Department of Pathogenic Biology and Immunology, Xuzhou Medical University, Xuzhou 221004, China; Jiangsu Key Laboratory of Immunity and Metabolism, Department of Pathogenic Biology and Immunology, Xuzhou Medical University, Xuzhou 221004, China; Jiangsu Key Laboratory of Immunity and Metabolism, Department of Pathogenic Biology and Immunology, Xuzhou Medical University, Xuzhou 221004, China; Jiangsu Key Laboratory of Immunity and Metabolism, Department of Pathogenic Biology and Immunology, Xuzhou Medical University, Xuzhou 221004, China; Jiangsu Key Laboratory of Immunity and Metabolism, Department of Pathogenic Biology and Immunology, Xuzhou Medical University, Xuzhou 221004, China; Jiangsu Key Laboratory of Immunity and Metabolism, Department of Pathogenic Biology and Immunology, Xuzhou Medical University, Xuzhou 221004, China; Jiangsu Key Laboratory of Immunity and Metabolism, Department of Pathogenic Biology and Immunology, Xuzhou Medical University, Xuzhou 221004, China; Jiangsu Key Laboratory of Immunity and Metabolism, Department of Pathogenic Biology and Immunology, Xuzhou Medical University, Xuzhou 221004, China; National Demonstration Center for Experimental Basic Medical Sciences Education, Xuzhou Medical University, Xuzhou 221004, China; Jiangsu Key Laboratory of Immunity and Metabolism, Department of Pathogenic Biology and Immunology, Xuzhou Medical University, Xuzhou 221004, China; National Demonstration Center for Experimental Basic Medical Sciences Education, Xuzhou Medical University, Xuzhou 221004, China

**Keywords:** hepatocellular carcinoma, HBX, GLUD1, LASP1, SYVN1

## Abstract

Glutamate dehydrogenase 1 (GLUD1) is implicated in oncogenesis. However, little is known about the relationship between GLUD1 and hepatocellular carcinoma (HCC). In the present study, we demonstrated that the expression levels of GLUD1 significantly decreased in tumors, which was relevant to the poor prognosis of HCC. Functionally, GLUD1 silencing enhanced the growth and migration of HCC cells. Mechanistically, the upregulation of interleukin-32 through AKT activation contributes to GLUD1 silencing-facilitated hepatocarcinogenesis. The interaction between GLUD1 and AKT, as well as α-ketoglutarate regulated by GLUD1, can suppress AKT activation. In addition, LIM and SH3 protein 1 (LASP1) interacts with GLUD1 and induces GLUD1 degradation via the ubiquitin–proteasome pathway, which relies on the E3 ubiquitin ligase synoviolin (SYVN1), whose interaction with GLUD1 is enhanced by LASP1. In hepatitis B virus (HBV)-related HCC, the HBV X protein (HBX) can suppress GLUD1 with the participation of LASP1 and SYVN1. Collectively, our data suggest that GLUD1 silencing is significantly associated with HCC development, and LASP1 and SYVN1 mediate the inhibition of GLUD1 in HCC, especially in HBV-related tumors.

## Introduction

Hepatocellular carcinoma (HCC) is a high-mortality malignancy. Among the etiologies of HCC, hepatitis B virus (HBV) is a predominant risk factor (Harputluoglu and Carr, 2021; Ito and Nguyen, 2023). In particular, the HBV X protein (HBX), a regulatory protein encoded by the virus, can change the transcription of various genes and activate multiple signal transduction pathways to modulate the growth, metastasis, differentiation, and autophagy of HCC cells (Liu et al., 2016; Yang et al., 2022; Schollmeier et al., 2023). Because HBX plays a leading role in hepatocarcinogenesis, the viral protein and associated cellular factors are considered potential targets for HCC treatment (Medhat et al., 2021). Further investigation into the mechanisms underlying HBX-induced hepatocarcinogenesis could help us develop specific targeted treatments for HBV-related tumors.

Glutamate dehydrogenase 1 (GLUD1) is an important enzyme that converts glutamate to α-ketoglutarate (α-KG) (Maus and Peters, 2017; Yuzaki and Aricescu, 2017). Previous studies indicated that GLUD1 is involved in the development of glioblastoma, lung cancer, breast cancer, and kidney cancer (Jin et al., 2015; Yang et al., 2020; Shao et al., 2022) and affects cellular growth, invasion, and glutamine metabolism. However, discrepant expression patterns of this protein were observed in different cancers. For instance, GLUD1 expression is elevated in lung and breast cancers (Jin et al., 2015; Shao et al., 2022) but decreases in clear cell renal cell carcinoma (Wang et al., 2022). The role of GLUD1 in HCC, in particular HBV-induced HCC, remains unclear.

LIM and SH3 protein 1 (LASP1) is a multifunctional cytoskeletal protein that modulates cell growth, migration, signal transduction, and invasion by interacting with its binding partners to facilitate the progression of cancers (Hu et al., 2017; Butt and Raman, 2018), including breast, colorectal, and bladder cancers. We previously demonstrated that LASP1 regulates the development of HBV-associated HCC, primarily controlled by HBX (You et al., 2018, 2021, 2023a). However, the mechanisms underlying LASP1-induced hepatocarcinogenesis have not been elucidated.

Synoviolin (SYVN1) is an E3 ubiquitin ligase that modulates various biological functions by controlling the stability of proteins via a ubiquitin-dependent pathway (Liu et al., 2020a; Guo et al., 2021). SYVN1 is known to be relevant to HCC progression (Liu et al., 2018), although the underlying mechanisms are not fully understood. Here, we assessed the expression pattern and biological roles of GLUD1 in HCC and revealed novel mechanisms for LASP1 and SYVN1 to mediate HBX-induced inhibition of GLUD1 expression in HBV-related HCC. Thus, GLUD1 and its regulators may be utilized as potential drug targets for treating HCC, especially for HBV-induced malignancies.

## Results

### The expression and clinical association of GLUD1 in HCC

**Figure 1:**
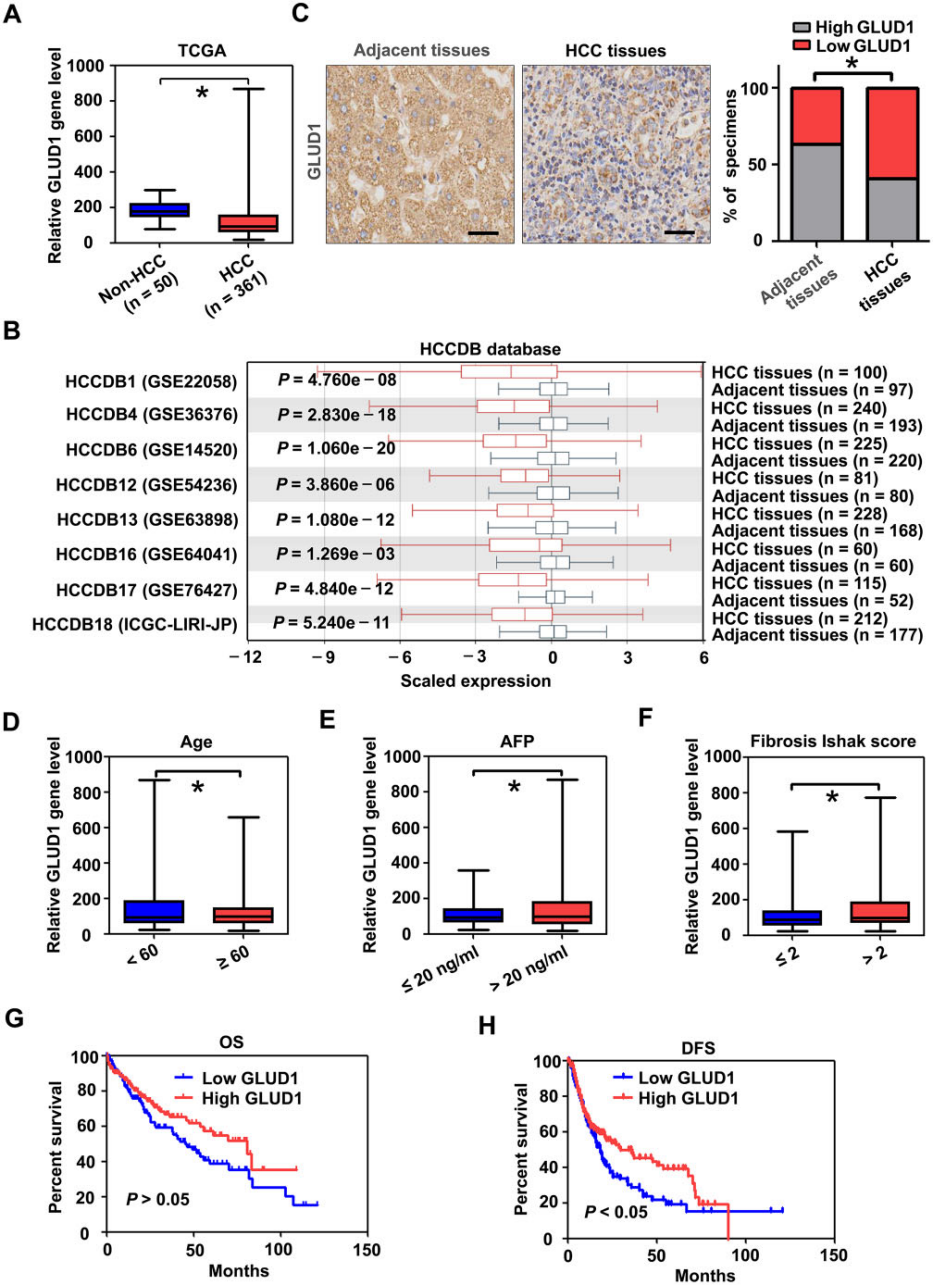
The expression and clinical association of GLUD1 in HCC. (**A**) The gene expression levels of GLUD1 in the TCGA HCC cohort. Non-HCC, normal liver tissues; HCC, HCC tissues. (**B**) The expression levels of GLUD1 in eight different cohorts from the HCCDB database. (**C**) The expression of GLUD1 in HCC tissues and adjacent tissues was detected by IHC. Scale bar, 50 μm. (**D**–**F**) The gene expression levels of GLUD1 associated with the age (**D**), AFP level (**E**), and fibrosis Ishak score (**F**) of HCC samples in the TCGA cohort. (**G** and **H**) The OS (**G**) and DFS (**H**) associated with low and high expression of GLUD1 in HCC through univariate survival analysis. *P < 0.05.

To clarify the association between GLUD1 expression and HCC progression, we assessed GLUD1 levels in the tumors using several databases. As shown in Figure 1A, the gene levels of GLUD1 were lower in HCC tissues than in normal liver tissues in the TCGA database (Hu et al., 2022). An analysis of eight HCC cohorts from the HCCDB database (Lian et al., 2018) confirmed the downregulation of GLUD1 in the tumors (Figure 1B). Consistently, immunohistochemistry (IHC) analysis of tissue samples collected by our group demonstrated that the expression of GLUD1 was significantly lower in HCC tissues than in adjacent normal tissues (Figure 1C). Based on TCGA cohorts, correlations between various clinical factors and GLUD1 expression were assessed. As shown in Figure 1D–F, the gene expression levels of GLUD1 were lower in HCC patients aged < 60 years (compared to ≥60 years), with the alpha-fetoprotein (AFP) level ≤20 ng/ml (compared to >20 ng/ml), and with a fibrosis Ishak score ≤2 (compared to >2). Univariate survival analysis revealed that GLUD1 silencing was significantly associated with unfavorable decrease-free survival (DFS), but not significantly with overall survival (OS), in patients with HCC (Figure 1G and H). Altogether, these observations suggest that the reduced expression of GLUD1 is relevant to HCC development and poor tumor prognosis.

### GLUD1 regulates the growth and migration of HCC cells

Next, we examined the biological functions of GLUD1 in HCC cells. The HA-labeled GLUD1 expression plasmid was constructed and transfected into the tumor cell lines HepG2 and Huh7 (Supplementary Figure S1A). Overexpression of GLUD1 dramatically suppressed the proliferation of HCC cells (Supplementary Figure S1B and C) and HCC cell migration *in vitro* (Supplementary Figure S1D and E). The tumor xenograft assay in nude mice determined the effect of GLUD1 overexpression on the suppression of HCC cell proliferation *in vivo* (Supplementary Figure S1F–H). Meanwhile, specific short hairpin RNA (shRNA) targeting GLUD1 was transfected into HepG2 and Huh7 cells (Supplementary Figure S1I). GLUD1 silencing enhanced HCC cell growth (Supplementary Figure S1J and K) and migration (Supplementary Figure S1L and M) *in vitro* and the growth of HCC tumor xenografts *in vivo* (Supplementary  Figure S1N–P).

### GLUD1 controls interleukin-32 expression in HCC

**Figure 2:**
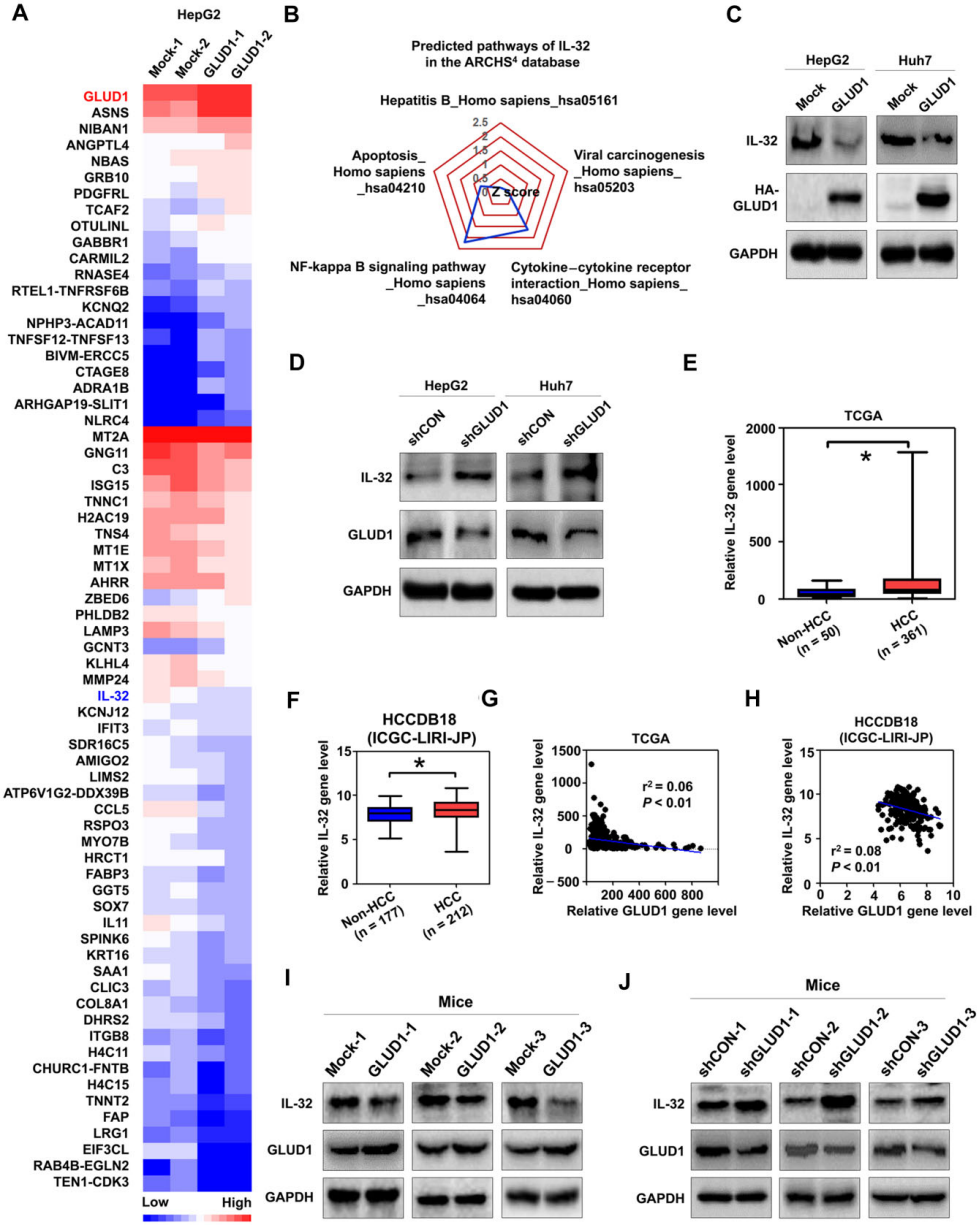
The expression of IL-32 is regulated by GLUD1 in HCC. (**A**) Differential genes were determined by RNA sequencing in HepG2 cells with or without exogenous GLUD1 gene expression. Mock, HCC cells transfected with control expression plasmids; GLUD1, HCC cells transfected with exogenous HA-labeled GLUD1. (**B**) Predicted pathways associated with IL-32 based on the ARCHS^4^ database. (**C** and **D**) Effects of exogenous GLUD1 (**C**) and GLUD1 silencing (**D**) on the protein expression of IL-32 in HCC cells. HA-GLUD1, HA-labeled GLUD1 protein; shCON, HCC cells transfected with control shRNA; shGLUD1, HCC cells transfected with shRNA targeting GLUD1. (**E** and **F**) The gene expression levels of IL-32 in the TCGA HCC cohort (**E**) and the ICGC-LIRI-JP cohort (**F**). (**G** and **H**) Correlations between GLUD1 and IL-32 gene expression levels in HCC tissues in the TCGA cohort (**G**) and the ICGC-LIRI-JP cohort (**H**). (**I** and **J**) Effects of GLUD1 overexpression (**I**) and GLUD1 inhibition (**J**) on the protein expression of IL-32 in tumor xenografts. *P < 0.05.

To investigate the downstream molecules of GLUD1, RNA sequencing was performed on HepG2 cells with or without exogenous GLUD1 gene expression, and 21 upregulated genes and 47 downregulated genes controlled by GLUD1 were identified (Figure 2A). Among the downregulated genes, interleukin-32 (IL-32) was chosen for further study, because the cytokine IL-32 has been demonstrated to facilitate the growth and migration of HCC cells, and the increase of IL-32 in HCC cells is relevant to HBV infection (Pan et al., 2011; Kang et al., 2012; Zhao et al., 2018). Besides, based on the ARCHS^4^ database (Lachmann et al., 2018), IL-32 was predicted to be involved in hepatitis B, cytokine–cytokine receptor interaction, and viral carcinogenesis pathways (Figure 2B). Furthermore, overexpression of exogenous GLUD1 inhibited IL-32 protein expression (Figure 2C), whereas GLUD1 silencing by shRNA promoted IL-32 protein expression (Figure 2D) in HCC cells. Analyses based on the TCGA and Japan Project of International Cancer Genome Consortium (ICGC-LIRI-JP) HCC cohorts (Hu et al., 2022) revealed significantly higher gene expression levels of IL-32 in HCC tissues compared to noncancerous tissues (Figure 2E and F) and a negative correlation between GLUD1 and IL-32 gene expression levels in HCC tissues from either cohort (Figure 2G and H). Moreover, GLUD1 inhibited the protein expression of IL-32 in HCC tumor xenografts (Figure 2I and J; Supplementary Figure S1F and N).

### AKT activation mediates IL-32 expression regulated by GLUD1 in HCC

**Figure 3:**
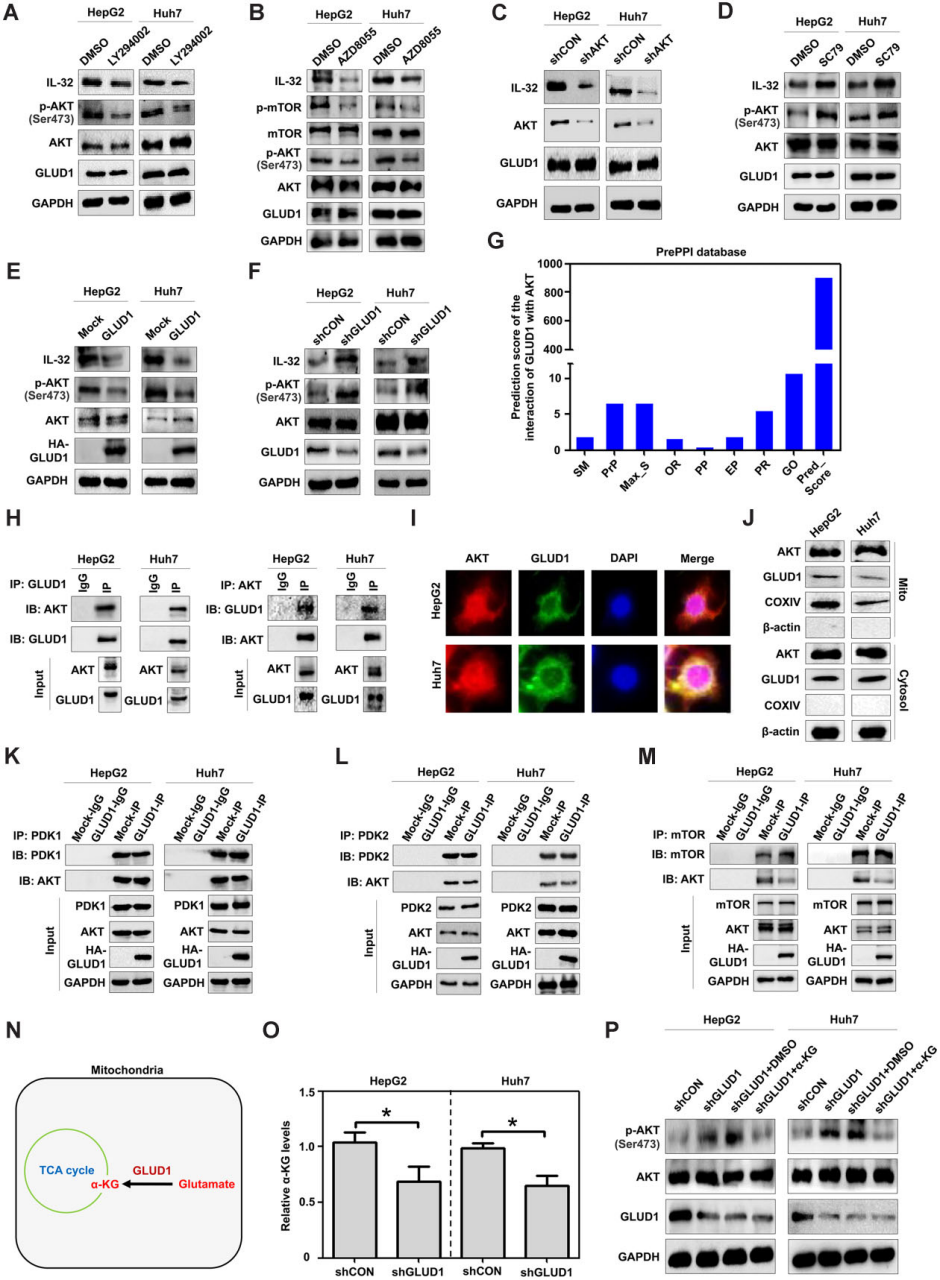
AKT activation mediates IL-32 expression regulated by GLUD1 in HCC cells. (**A**–**F**) Effects of the PI3K pathway inhibitor LY294002 (**A**), the mTOR inhibitor AZD8055 (**B**), AKT knockdown (**C**), the AKT activator SC79 (**D**), exogenous GLUD1 (**E**), and GLUD1 silencing (**F**) on the protein expression of IL-32 in HCC cells. (**G**) Prediction of GLUD1 interaction with potential proteins based on the PrePPI database suggests an interaction between GLUD1 and AKT. (**H**) The interaction between GLUD1 and AKT was detected by co-IP. (**I**) The co-localization of GLUD1 with AKT in HCC cells was determined by immunofluorescence. (**J**) The protein expression of GLUD1 and AKT in the mitochondria and cytosol (extracted using the Mitochondria/Cytosol Protein Isolation Kit) of HCC cells was detected by western blotting. (**K**–**M**) Effects of GLUD1 on the interactions between AKT and PDK1 (**K**), PDK2 (**L**), and mTOR (**M**) in HCC cells. (**N**) Sketch map showing the role of GLUD1 in the production of α-KG in mitochondria. (**O**) Effects of GLUD1 silencing on the production of α-KG in HCC cells. (**P**) Effects of GLUD1 silencing in the absence or presence of α-KG on the activation of AKT in HCC cells. *P < 0.05.

Previous studies reported that activation of AKT in the PI3K signaling pathway contributes to IL-32 upregulation in different cells (Nishida et al., 2008; Li et al., 2015). In addition, both PDK1/2 and mTOR could activate AKT (Vara et al., 2004; Sarbassov et al., 2005). Here, both the PI3K pathway inhibitor LY294002 and the mTOR inhibitor AZD8055 repressed AKT phosphorylation and decreased IL-32 levels (Figure 3A and B), suggesting that AKT activation participates in the regulation of IL-32 expression in HCC cells. The knockdown of AKT by its specific shRNA could suppress IL-32 levels (Figure 3C), while activating AKT via SC79 promoted its expression (Figure 3D), confirming that the activation of AKT was responsible for the upregulation of IL-32 in HCC cells. Meanwhile, either activating or inhibiting AKT did not obviously affect the protein expression of GLUD1 (Figure 3A–D). However, GLUD1 overexpression inhibited AKT phosphorylation at Ser473 and suppressed IL-32 levels (Figure 3E), while GLUD1 silencing promoted AKT phosphorylation and IL-32 levels (Figure 3F), suggesting that the inhibition of AKT activation is involved in the suppression of IL-32 by GLUD1 in HCC cells.

Using the PrePPI database (Zhang et al., 2013), we identified that AKT had the potential to interact with GLUD1, based on the different predicted pathways with diverse scores (Figure 3G). The interaction between GLUD1 and AKT in HCC cells was validated by co-immunoprecipitation (co-IP) (Figure 3H). The co-localization of GLUD1 and AKT was demonstrated by immunofluorescence (Figure 3I). Although GLUD1 is a mitochondrial enzyme, it can be located in the mitochondria or cytoplasm in different types of cells (Spanaki et al., 2017). Using a Mitochondria/Cytosol Protein Isolation Kit, followed by western blot analysis, GLUD1 and AKT proteins were detected in both the mitochondria and cytosol of HCC cells (Figure 3J), suggesting that GLUD1 interacts with AKT and affects AKT activation directly in these regions of tumor cells.

Since the activation of AKT relies on PDK1/2 and mTOR (Vara et al., 2004; Sarbassov et al., 2005), we speculated that GLUD1 may affect the binding between AKT and PDK1/2 or mTOR to inhibit AKT activation. Co-IP assays demonstrated that the interaction between mTOR and AKT decreased, while the interaction between PDK1/2 and AKT did not change in GLUD1-overexpressing HepG2 and Huh7 cells compared with control cells (Figure 3K–M), suggesting that the inhibition of AKT activation mediated by GLUD1 is closely related to the suppression of the interaction between mTOR and AKT.

In addition, GLUD1 is an important enzyme that can convert glutamate to α-KG (Yuzaki and Aricescu, 2017; Figure 3N). After GLUD1 inhibition, α-KG levels in HepG2 and Huh7 cells were suppressed (Figure 3O). A study by Shrimali et al. (2021) indicated that α-KG inhibits AKT activation. We observed that the enhanced AKT phosphorylation after GLUD1 silencing in HCC cells were reduced by incubation with exogenous α-KG (Figure 3P). These data suggested that α-KG suppression, mediated by GLUD1 silencing, facilitates the activation of AKT to modulate the PI3K signaling pathway.

### LASP1 mediates the inhibition of GLUD1 in HCC

**Figure 4:**
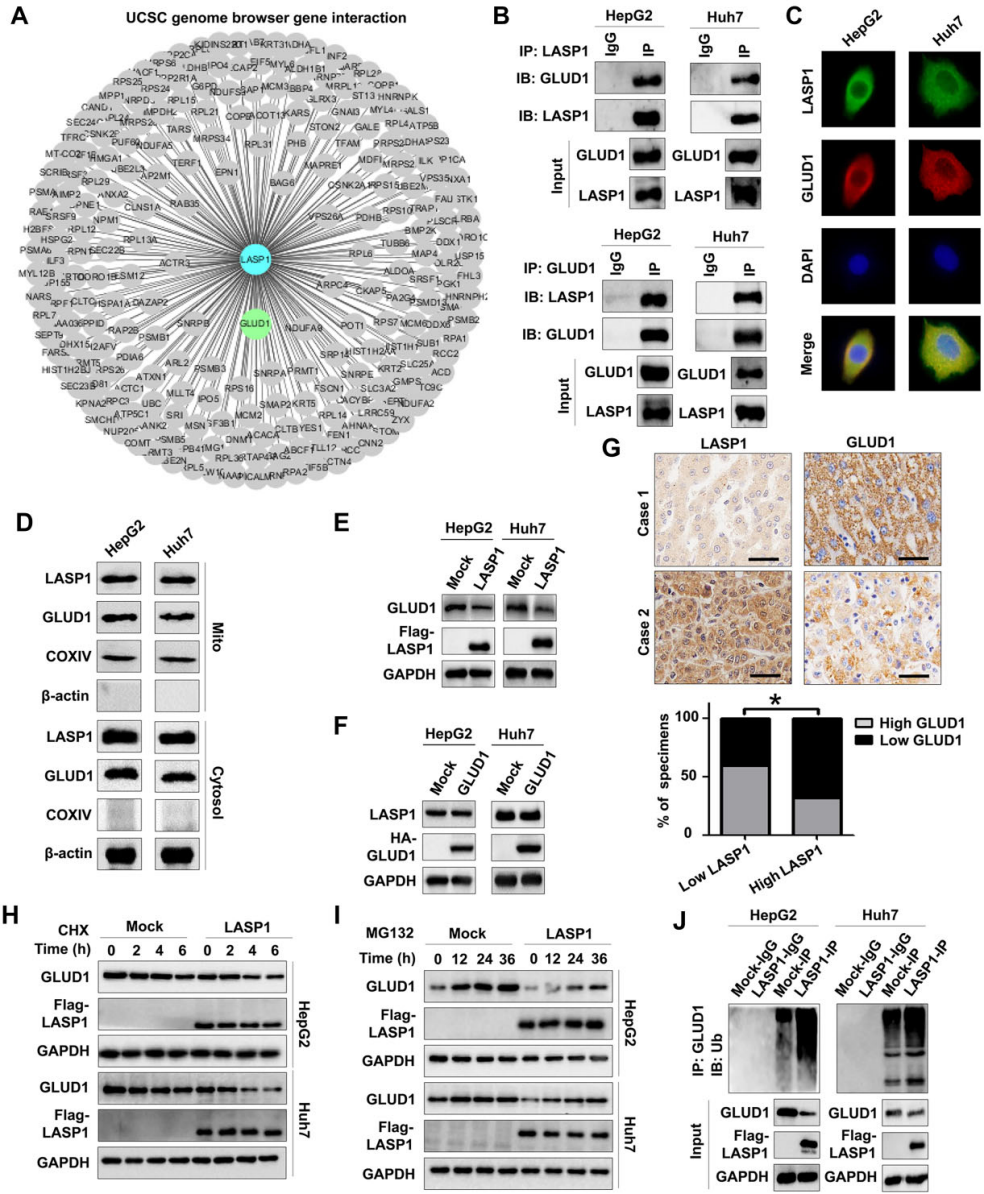
LASP1 suppresses GLUD1 protein expression in HCC. (**A**) The predicted interaction between LASP1 and GLUD1 based on the UCSC genome browser gene interaction database. (**B**) The interaction between LASP1 and GLUD1 was detected by co-IP. (**C**) The co-localization of LASP1 with GLUD1 in HCC cells was determined by immunofluorescence. (**D**) The protein expression of GLUD1 and LASP1 in the mitochondria and cytosol of HCC cells was detected by western blotting. (**E**) Effects of exogenous LASP1 on GLUD1 protein expression in HCC cells. LASP1, HCC cells transfected with Flag-labeled LASP1; Flag-LASP1, Flag-labeled LASP1 protein. (**F**) Effects of exogenous GLUD1 on LASP1 protein expression in HCC cells. (**G**) Correlations between LASP1 and GLUD1 expression in HCC tissues detected by IHC. Scale bar, 50 μm. (**H** and **I**) GLUD1 protein levels in exogenous LASP1-overexpressing and control HCC cells treated with 200 μg/ml CHX (**H**) or 100 nM MG132 (**I**) for the indicated periods. (**J**) Effects of LASP1 on GLUD1 ubiquitination in HCC cells. Ub, ubiquitin. *P < 0.05.

We subsequently determined the cellular factors contributing to the inhibition of GLUD1 in HCC. LASP1 is a cytoskeletal protein that facilitates HCC development (Wang et al., 2009; Salvi et al., 2015; You et al., 2021, 2023a). Using the UCSC genome browser gene interaction database (Mangan et al., 2014), we predicted the interaction between GLUD1 and LASP1 (Figure 4A). Then, the interaction and co-localization of GLUD1 with LASP1 in HCC cells were demonstrated by Co-IP and immunofluorescence assays (Figure 4B and C). LASP1 and GLUD1 were also detected in both the mitochondria and cytosol (Figure 4D), indicating that LASP1 can bind to GLUD1 in these regions of HCC cells. Furthermore, LASP1 overexpression suppressed GLUD1 expression in HepG2 and Huh7 cells (Figure 4E), whereas GLUD1 did not significantly affect LASP1 expression in HCC cells (Figure 4F). IHC analyses for LASP1 and GLUD1 expression in HCC tissues revealed a negative correlation between their protein levels (Figure 4G). Since LASP1 can affect its target protein stability by modulating ubiquitination (You et al., 2021), we examined GLUD1 ubiquitination and stability in HCC cells by using the protein synthesis inhibitors cycloheximide (CHX) and MG132 (a proteasome inhibitor). LASP1 facilitated the degradation of GLUD1 protein (Figure 4H and I), which was related to the upregulation of GLUD1 ubiquitination mediated by LASP1 (Figure 4J).

### LASP1-mediated inhibition of GLUD1 relies on SYVN1 in HCC

**Figure 5:**
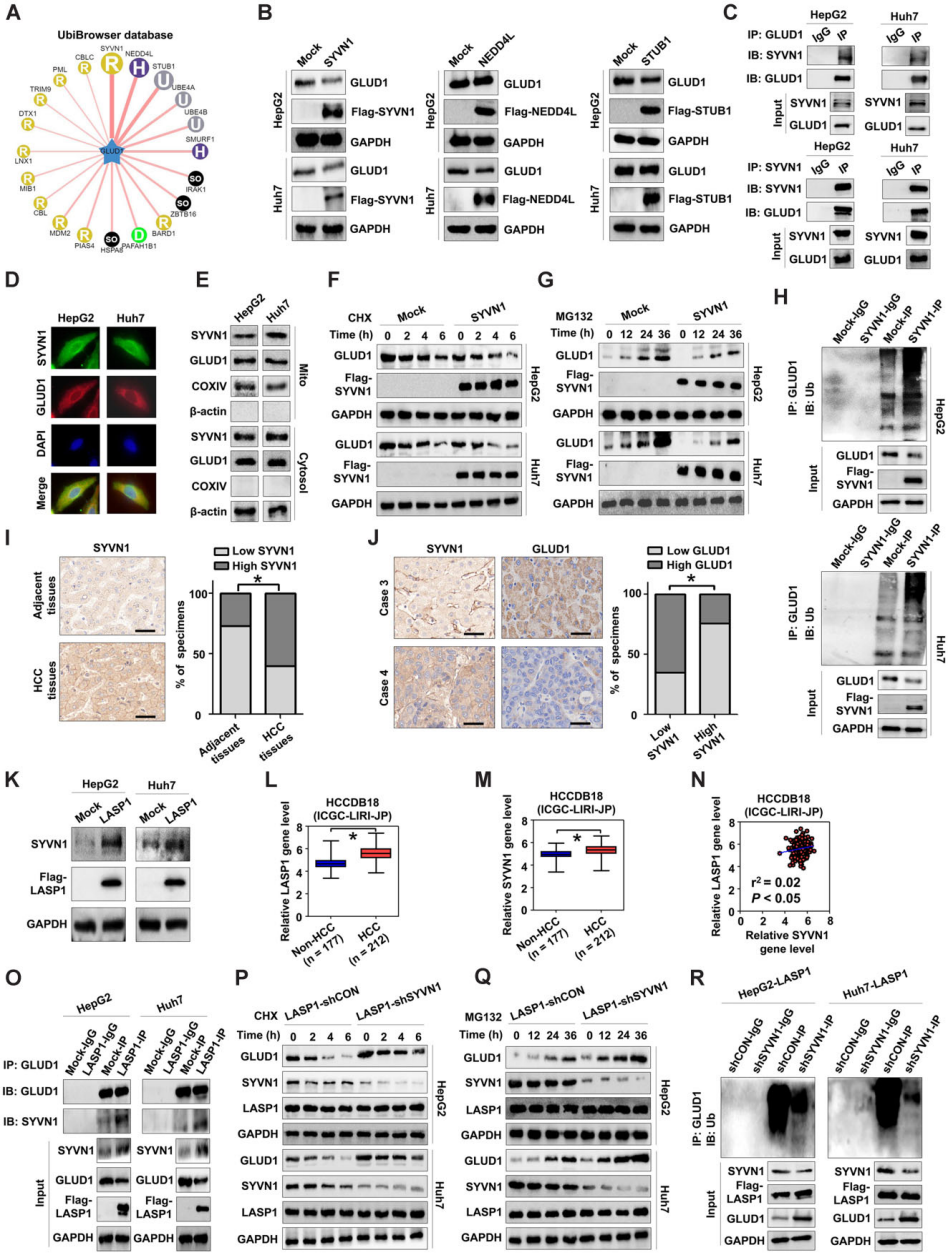
LASP1-mediated inhibition of GLUD1 is associated with SYVN1 in HCC. (**A**) Predicted E3 ligases of GLUD1 based on the UbiBrowser database. (**B**) Effects of SYVN1, NEDD4L, and STUB1 on the protein expression of GLUD1 in HCC cells. SYVN, HCC cells transfected with Flag-labeled SYVN1; NEDD4L, HCC cells transfected with Flag-labeled NEDD4L; STUB1, HCC cells transfected with Flag-labeled STUB1; Flag-SYVN1, Flag-labeled SYVN1 protein; Flag-NEDD4L, Flag-labeled NEDD4L protein; Flag-STUB1, Flag-labeled STUB1 protein. (**C**) The interaction between GLUD1 and SYVN1 was detected by co-IP. (**D**) The co-localization of GLUD1 with SYVN1 in HCC cells was determined by immunofluorescence. (**E**) The protein expression of GLUD1 and LASP1 in the mitochondria and cytosol of HCC cells was detected by western blotting. (**F** and **G**) GLUD1 protein levels in exogenous SYVN1-overexpressing and control HCC cells treated with 200 μg/ml CHX (**F**) or 100 nM MG132 (**G**) for the indicated periods. (**H**) Effects of SYVN1 on GLUD1 ubiquitination in HCC cells. (**I**) The expression of SYVN1 in HCC tissues and adjacent normal tissues was detected by IHC. Scale bar, 50 μm. (**J**) Correlations between GLUD1 and SYVN1 expression in HCC tissues detected by IHC. Scale bar, 50 μm. (**K**) Effects of exogenous LASP1 on SYVN1 protein expression in HCC cells. (**L**–**N**) The gene expression levels of LASP1 (**L**) and SYVN1 (**M**) and their correlations (**N**) in HCC tissues from the ICGC-LIRI-JP cohort. (**O**) Effects of LASP1 on the interaction between SYVN1 and GLUD1 in HCC cells. (**P** and **Q**) Effects of SYVN1 silencing on the protein expression of GLUD1 in exogenous LASP1-overexpressing HCC cells treated with 200 μg/ml CHX (**P**) or 100 nM MG132 (**Q**) for the indicated periods. shSYVN1, HCC cells transfected with shRNA targeting SYVN1. (**R**) Effects of SYVN1 silencing on GLUD1 ubiquitination mediated by LASP1 in HCC cells. *P < 0.05.

Since LASP1 is not an E3 ligase (Kong et al., 2019a) that directly modulates GLUD1 ubiquitination, we then determined E3 ligases of GLUD1. Using the UbiBrowser database (version 1.0) (Li et al., 2017), we predicted several potential E3 ligases, including SYVN1, NEDDL4, and STUB1 (Figure 5A), but only SYVN1 was found to significantly inhibit GLUD1 protein levels in HCC cells (Figure 5B). The interaction and co-localization of SYVN1 with GLUD1 were also demonstrated (Figure 5C and D). Both SYVN1 and GLUD1 were detected in the mitochondria and cytosol (Figure 5E), indicating that SYVN1 binds to GLUD1 in these regions of HCC cells. SYVN1 induced suppression of GLUD1 protein stability in HCC cells (Figure 5F and G), which was related to the elevated GLUD1 ubiquitination (Figure 5H). In addition, IHC analyses revealed higher SYVN1 expression levels in HCC tissues compared to adjacent normal tissues (Figure 5I) and a negative correlation between SYVN1 and GLUD1 protein levels in HCC tissues (Figure 5J).

We next assessed whether the regulation of GLUD1 ubiquitination and stability by LASP1 relies on SYVN1. We found that LASP1 could enhance SYVN1 protein levels in HepG2 and Huh7 cells (Figure 5K). The gene expression levels of both LASP1 and SYVN1 were higher in HCC tissues than in nontumorous tissues from the ICGC-LIRI-JP cohort (Figure 5L and M), and there was a positive correlation between LASP1 and SYVN1 gene levels in the HCC cohort (Figure 5N). In addition, based on the UbiBrowser database, SYVN1 was determined as a potent E3 ligase of LASP1 with a low score (Supplementary Figure S2A). However, exogenous SYVN1 did not impact on LASP1 expression (Supplementary Figure S2B), though they did interact with each other (Supplementary Figure S2C). Then, we investigated whether LASP1 could promote the binding between SYVN1 and GLUD1 and found that compared to control cells, the interaction between GLUD1 and SYVN1 was enhanced in LASP1-overexpressing HCC cells (Figure 5O). Furthermore, SYVN1 silencing reversed the LASP1-induced suppression of GLUD1 protein stability (Figure 5P and Q) and upregulation of GLUD1 ubiquitination (Figure 5R) in LASP1-overexpressing HCC cells. Collectively, these findings support that the suppression of GLUD1 stability mediated by LASP1 relies on SYVN1 in HCC cells.

### HBX restrains GLUD1 expression in HBV-induced HCC

**Figure 6:**
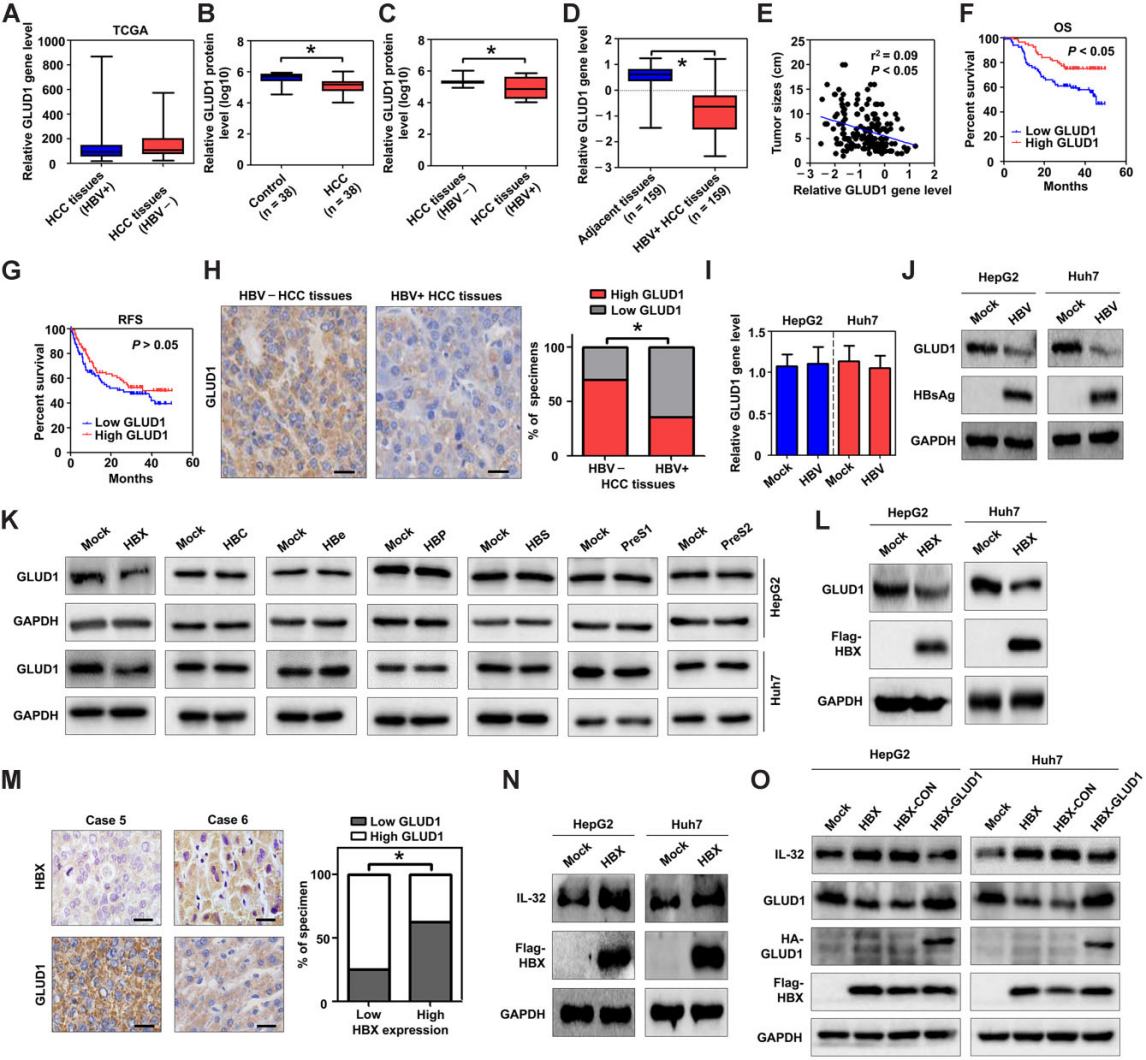
HBX restrains the protein expression of GLUD1 in HCC. (**A**) The gene expression levels of GLUD1 in HBV-negative (HBV–) and HBV-positive (HBV+) HCC tissues from the TCGA cohort. (**B** and **C**) The protein expression levels of GLUD1 in HCC tissues from the Zhu cohort. (**D**) The gene expression levels of GLUD1 in HBV+ HCC tissues and adjacent normal tissues from the Gao cohort. (**E**) Correlations between tumor size and GLUD1 gene expression level in HCC tissues from the Gao cohort. (**F** and **G**) The OS (**F**) and RFS (**G**) associated with low and high expression of GLUD1 in HBV-related HCC in the Gao cohort. (**H**) The expression of GLUD1 in HBV– and HBV+ HCC tissues was detected by IHC. Scale bar, 20 μm. (**I** and **J**) Effects of HBV on GLUD1 gene (**I**) and protein (**J**) expression in HCC cells were determined by real-time PCR and western blotting, respectively. HBV, HCC cells transfected with HBV expression plasmids. (**K** and **L**) The protein expression of GLUD1 in HCC cells transiently transfected with various viral proteins (**K**) or stably transfected with HBX (**L**) was detected by western blotting. HBX, HCC cells transfected with Flag-labeled HBX; Flag-HBX, Flag-labeled HBX protein. (**M**) Correlations between GLUD1 and HBX expression in HCC tissues detected by IHC. Scale bar, 20 μm. (**N**) Effects of HBX on IL-32 protein expression in HCC cells. (**O**) Effects of GLUD1 on the protein expression of IL-32 in HBX-expressing HCC cells. HBX-CON, HBX-expressing HCC cells transfected with control expression plasmids; HBX-GLUD1, HBX-expressing HCC cells transfected with HA-labeled GLUD1 expression plasmids. *P < 0.05.

HBV is a major etiological factor of HCC (Arslan et al., 2021). Next, we determined whether the virus could affect GLUD1 expression in HCC tissues. Based on the TCGA cohort, there was no significant difference in the gene expression levels of GLUD1 between HBV-negative and HBV-positive HCC tissues (Figure 6A). Based on the Zhu cohort (Zhu et al., 2019), GLUD1 protein levels were significantly lower in HCC tissues than in control tissues (Figure 6B) and also significantly lower in HBV-positive HCC tissues than in HBV-negative HCC tissues (Figure 6C). Based on the Gao cohort (Gao et al., 2019), GLUD1 gene levels were significantly lower in HBV-related HCC tissues than in adjacent tissues (Figure 6D), a negative correlation between GLUD1 gene level and tumor size in HBV-related HCC cohort was detected (Figure 6E), and low GLUD1 expression was significantly associated with poor OS, but not recurrence-free survival (RFS), in patients with HBV-related HCC (Figure 6F and G). IHC analyses further confirmed the lower expression of GLUD1 in HBV-positive HCC tissues than in HBV-negative HCC tissues (Figure 6H). In addition, in HCC cells transfected with HBV plasmids, HBV decreased GLUD1 protein levels, but not its gene levels (Figure 6I and J).

To date, various HBV-encoded proteins, including HBX (Kong et al., 2019b; You et al., 2023b), HBC (Kong et al., 2020), PreS1 (Liu et al., 2017), and PreS2 (Teng et al., 2015), have been reported to participate in HBV-mediated HCC. Our results showed that only HBX, either transiently or stably overexpressed in HCC cells, downregulated GLUD1 protein levels (Figure 6K and L). IHC analyses also revealed a negative correlation between HBX and GLUD1 expression levels in HCC tissues (Figure 6M). Besides, IL-32 protein levels were upregulated in HBX-expressing HCC cells (Figure 6N), confirming that IL-32 could be upregulated by HBX (Pan et al., 2011). However, after transfecting exogenous GLUD1 into HBX-expressing HCC cells, the viral protein-mediated upregulation of IL-32 was restricted (Figure 6O), suggesting that the inhibition of GLUD1 is involved in HBX-mediated IL-32 upregulation.

### LASP1 and SYVN1 mediate HBX-regulated GLUD1 stability

**Figure 7:**
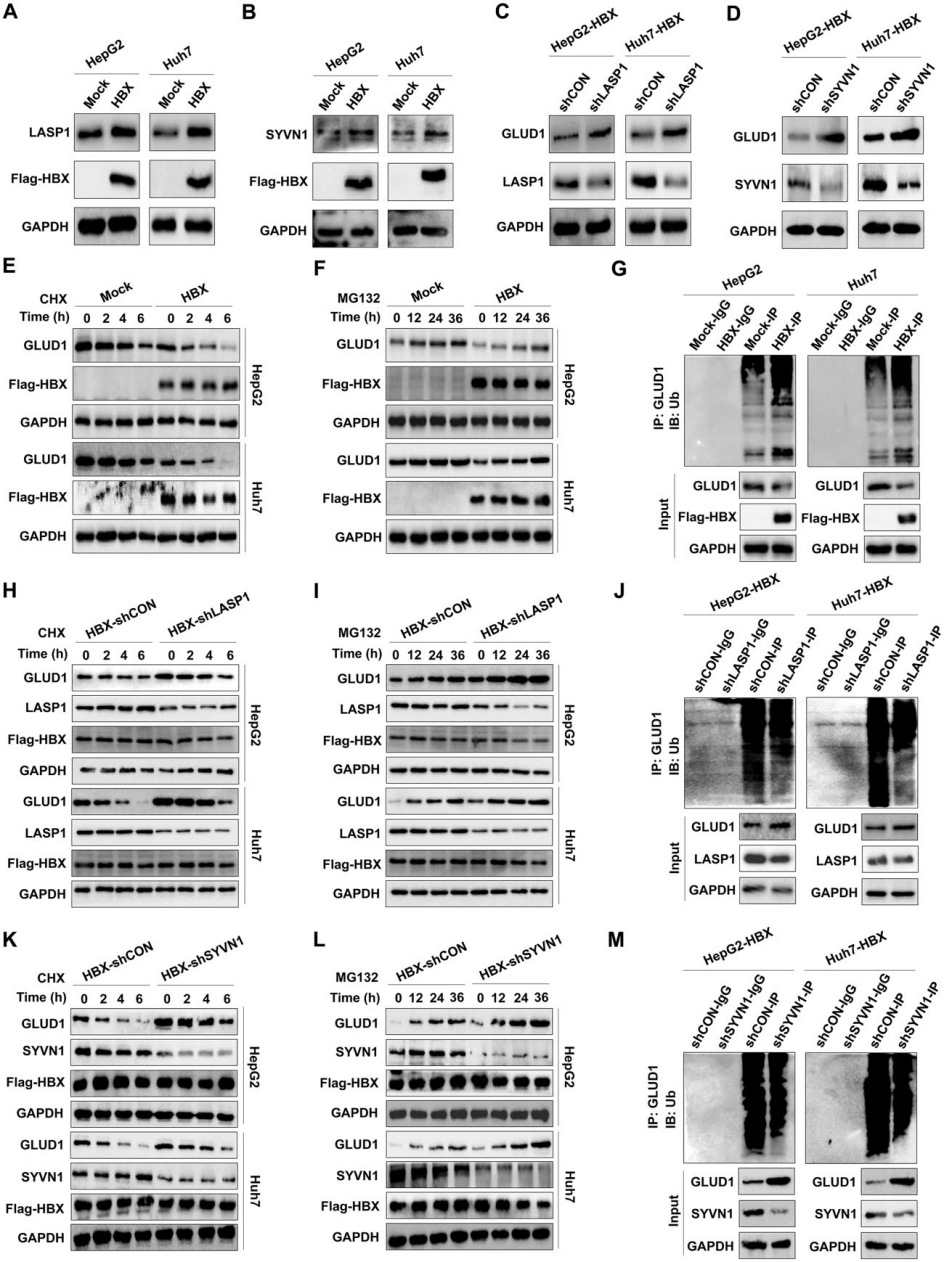
LASP1 and SYVN1 mediate the restraint of GLUD1 expression by HBX in HCC cells. (**A** and **B**) Effects of HBX on the protein expression of LASP1 (**A**) and SYVN1 (**B**) in HCC cells. (**C** and **D**) Effects of LASP1 silencing (**C**) and SYVN1 silencing (**D**) on GLUD1 protein expression in HBX-expressing HCC cells. (**E** and **F**) GLUD1 protein levels in HBX-expressing and control cells treated with 200 μg/ml CHX (**E**) or 100 nM MG132 (**F**) for the indicated periods. (**G**) Effects of HBX on GLUD1 ubiquitination in HCC cells. (**H** and **I**) Effects of LASP1 silencing on the protein expression of GLUD1 in HBX-expressing HCC cells treated with 200 μg/ml CHX (**H**) or 100 nM MG132 (**I**) for the indicated periods. (**J**) Effects of LASP1 silencing on GLUD1 ubiquitination in HBX-expressing HCC cells. (**K** and **L**) Effects of SYVN1 silencing on the protein expression of GLUD1 in HBX-expressing HCC cells treated with 200 μg/ml CHX (**K**) or 100 nM MG132 (**L**) for the indicated periods. (**M**) Effects of SYVN1 silencing on GLUD1 ubiquitination in HBX-expressing HCC cells.

We further assessed whether LASP1 and SYVN1 are involved in HBX-induced modulation of GLUD1 in HCC. As shown in Figure 7A and B, both LASP1 and SYVN1 protein levels increased in HBX-expressing HCC cells. Either LASP1 silencing or SYVN1 silencing by the specific shRNA enhanced the protein levels of GLUD1 in HBX-expressing HCC cells (Figure 7C and D). Furthermore, HBX reduced GLUD1 protein stability by promoting GLUD1 ubiquitination (Figure 7E–G), which was reversed by either LASP1 silencing or SYVN1 silencing in HBX-expressing HepG2 and Huh7 cells (Figure 7H–M). Collectively, these findings indicate that in HCC cells, LASP1 and SYVN1 mediate HBX-reduced GLUD1 stability in a ubiquitination-dependent manner.

### GLUD1 regulates the growth and migration of HBX-associated HCC cells

We also examined the effect of GLUD1 on the biological functions, i.e. growth and migration, of HBX-expressing HCC cells. Consistent with previous observations, overexpression of GLUD1 suppressed HBX-facilitated cell growth (Supplementary Figure S3A and B) and migration (Supplementary Figure S3C and D) in HBX-expressing HCC cells and the growth of HCC tumor xenografts induced by HBX *in vivo* (Supplementary Figure S3E–G).

## Discussion

HCC remains a challenging malignant disease owing to its poor prognosis (Ito and Nguyen, 2023). To date, the detailed molecular mechanisms underlying the emergence and development of HCC are not well understood. GLUD1 modulates the development of various cancers (Jin et al., 2015; Yang et al., 2020; Shao et al., 2022). However, little is known about the association between GLUD1 and HCC. We determined that GLUD1 expression decreases in tumors. GLUD1 silencing facilitates the proliferation and migration of HCC cells and upregulates IL-32 expression mediated by AKT in the PI3K signaling pathway. Furthermore, GLUD1 interacts with AKT and thus inhibits mTOR-mediated AKT activation. Additionally, both LASP1 and SYVN1 bind to GLUD1 and suppress GLUD1 stability in a ubiquitination-dependent manner. In HBV-associated HCC, HBX mediates the inhibition of GLUD1 expression. LASP1 and SYVN1 also participate in HBX-mediated GLUD1 suppression (Supplementary Figure  S4).

GLUD1 not only converts glutamate to α-KG but also regulates multiple signaling pathways and downstream genes to modulate the development of kidney cancer, glioblastoma, and lung cancer (Jin et al., 2015; Yang et al., 2020; Shao et al., 2022). Thus, targeting GLUD1 is an attractive anticancer strategy. However, it should be noted that the expression of GLUD1 in different neoplasms is diverse (Jin et al., 2015; Shao et al., 2022). Therefore, characterizing the regulatory mechanisms of GLUD1 in different tumors is important for the development of optimal treatment strategies. Based on different HCC cohorts, we demonstrated that GLUD1 was downregulated in tumors, which was closely associated with various clinical parameters and the prediction of unfavorable outcomes in patients with HCC. Marsico et al. (2021) showed that inhibiting GLUD1 with small interfering RNA can reduce the proliferation of HepG2 cells but promote the growth of hepatocytes *in vitro*. However, in our study, shRNA-mediated GLUD1 silencing enhanced the growth and migration of HepG2 and Huh7 cells. The difference in proliferation rate was not notably large but significant. This may be due to the small number of cells used and not long enough time for observation. In addition, GLUD1-induced suppression of cell proliferation and migration was demonstrated in GLUD1-overexpressing HepG2 and Huh7 cells and tumor xenografts in nude mice. Notably, our investigation of the biological role of GLUD1 was dependent on liver cancer lines. To date, whether GLUD1 silencing triggers the transformation of normal hepatocytes to tumor cells is still unknown, and further studies on this issue are required.

Among the candidate genes downstream of GLUD1 in HepG2 cells identified by RNA sequencing analysis (Figure 2A), we determined that GLUD1 inhibited the expression level of IL-32, a cytokine that plays a role in HCC development (Pan et al., 2011; Kang et al., 2012). Additional candidate genes, including MTIE (Liu et al., 2020b), AHRR (Liang et al., 2012), and CCL5 (Xu et al., 2022), were associated with HCC development. Whether these genes participate in the regulation of hepatocarcinogenesis mediated by GLUD1 remains to be assessed in future studies. It has been shown that activation of AKT in the PI3K signaling pathway can increase IL-32 levels in different cells (Nishida et al., 2008; Li et al., 2015). Here, we identified an interaction between GLUD1 and AKT and confirmed the inhibition of AKT activation mediated by GLUD1. Wang et al. (2022) showed that GLUD1 suppresses AKT activation in clear cell renal cell carcinoma, which was consistent with our findings, but they did not investigate the underlying molecular mechanisms. In the PI3K signaling pathway, AKT is activated by the protein kinases PDK1/2 and mTOR (Vara et al., 2004; Sarbassov et al., 2005). Interestingly, we found that GLUD1-inhibited AKT phosphorylation was dependent on the decreased interaction between AKT and mTOR but not PDK1/2. In addition, α-KG is a catalytic product of GLUD1 (Yuzaki and Aricescu, 2017) and has also been demonstrated to inhibit AKT activation (Shrimali et al., 2021). We observed that the inhibition of GLUD1 decreased α-KG levels in HCC cells, while adding α-KG to GLUD1-silenced HCC cells reduced AKT phosphorylation. Thus, different mechanisms are involved in GLUD1-mediated inhibition of AKT activation to control IL-32 expression. However, the detailed mechanism underlying the suppression of AKT by α-KG requires further studies.

Recent work has suggested that LASP1, a cytoskeletal protein, promotes HCC cell proliferation, migration, and metastasis to facilitate hepatocarcinogenesis (Salvi et al., 2009; Wang et al., 2009; Tang et al., 2012, 2013; You et al., 2021, 2023a). Here, we determined that LASP1 inhibited GLUD1 expression in HCC cells. LASP1-mediated regulation of PTEN (Gao et al., 2018), S100A11 (Niu et al., 2016), and vimentin (Salvi et al., 2015; You et al., 2021) relies on its interactions with these molecules. Protein‒protein interactions can affect ubiquitination–proteasome-mediated protein degradation (Kong et al., 2019a). Here, we demonstrated that LASP1 binds to GLUD1 and enhances GLUD1 ubiquitination to reduce its stability and thus protein level in HCC cells.

Importantly, SYVN1, a key regulator of the pathogenesis of different tumors (Liu et al., 2018; Guo et al., 2021; Ji et al., 2021), was identified as a novel E3 ligase of GLUD1. Functionally, SYVN1 can decrease the protein stability of GLUD1 by promoting its ubiquitination. We observed that LASP1 enhances SYVN1 expression in HCC cells, although the mechanism is unknown. Interestingly, LASP1 was shown to interact with both SYVN1 and GLUD1 and act as an adaptor to enhance GLUD1 ubiquitination by increasing the binding between SYVN1 and GLUD1. These findings reveal a new mechanism that contributes to the reduction in GLUD1 stability to promote HCC progression.

HBV infection is an HCC risk factor (Arslan et al., 2021). The significance of HBX in triggering the transformation of normal hepatocytes to cancer cells and promoting the development of HBV-positive liver cancer has been studied (Neuveut et al., 2010; Medhat et al., 2021), but whether HBX regulates GLUD1 to promote hepatocarcinogenesis remains to be determined. We found that HBX facilitated GLUD1 downregulation in HBV-related HCC. The inhibition of GLUD1 contributed to an increase in IL-32 level in HBX-expressing cells and the increased HCC cell growth and migration caused by HBX *in vitro* and *in vivo*.

In a previous study, we reported that LASP1 can be elevated by HBX through the activation of c-Jun (You et al., 2018). Here, we demonstrated that LASP1 and SYVN1 were upregulated in HBX-expressing HCC cells, and HBX-facilitated GLUD1 inhibition relied on LASP1 and SYVN1. Collectively, these findings highlight the significance of LASP1 and SYVN1 in modulating HBX-mediated GLUD1 inhibition.

In this work, based on various cohorts, we assessed the correlations between GLUD1 with IL-32 gene expression levels in HCC tissues (Figure 2G and H), between SYVN1 and LASP1 gene expression levels in HCC tissues (Figure 5N), and between GLUD1 gene expression level and tumor size in HBV-associated HCC (Figure 6E). Although significant positive or negative statistics (*P* < 0.05) were found in these analyses, the Pearson r values were very small, implying that the linear association between these targets was not strong. To better confirm the linear association of these results, future investigations based on a larger number of HCC tissues, including HBV-positive HCC tissues, are required.

In conclusion, our study verified that GLUD1 expression is significantly downregulated in HCC, which facilitates the development of HCC by increasing IL-32 induced by the activation of AKT. Moreover, we presented novel mechanisms for LASP1 and SYVN1 to be involved in HBX-mediated GLUD1 inhibition in HBV-induced liver cancer. Altogether, our findings contribute to a better understanding of the role of GLUD1 and its regulatory mechanisms in HCC and highlight the clinical and biological basis for developing GLUD1 as a novel diagnostic and therapeutic marker for tumors, especially HBV-related HCC.

## Materials and methods

Materials regarding reagents, cell culture, immunofluorescence analysis, and other information are provided in Supplementary material.

## Supplementary Material

mjae014_Supplemental_File
