# Supplementary material for: Inhibition of GLUD1 mediated by LASP1 and SYVN1 contributes to hepatitis B virus X protein-induced hepatocarcinogenesis
Source: J Mol Cell Biol. 2024 Apr 8;16(4):mjae014. doi: 10.1093/jmcb/mjae014 (PMC11440430; doi:10.1093/jmcb/mjae014)
Supplement: mjae014_Supplemental_File [file mjae014_supplemental_file.pdf]

Supplementary material

Inhibition of GLUD1 mediated by LASP1 and SYVN1 contributes to hepatitis B virus X protein-induced hepatocarcinogenesis

Hong-Juan You[1,†], Qi Li[1,2,†], Li-Hong Ma[1], Xing Wang[1], Huan-Yang Zhang[1], Yu-Xin Wang[1], En-Si Bao[1], Yu-Jie Zhong[1], De-Long Kong[1], Xiang-Ye Liu[1], Fan-Yun Kong[1,*], Kui-Yang Zheng[1,3], and Ren-Xian Tang[1,3,*]

[1] Jiangsu Key Laboratory of Immunity and Metabolism, Department of Pathogenic Biology and Immunology, Xuzhou Medical University, Xuzhou 221004, China

[2] Laboratory Department, The People's Hospital of Funing, Yancheng 224400, China

[3] National Demonstration Center for Experimental Basic Medical Sciences Education, Xuzhou Medical University, Xuzhou 221004, China

[†] These authors contributed equally to this work.

* Correspondence to: Ren-Xian Tang, E-mail: tangrenxian-t@163.com; Fan-Yun Kong, E-mail: kong.fanyun@163.com

Supplementary Materials and methods

Reagents and cell culture

Matrigel solution; MG132; G418; LASP1 shRNA plasmid; Flag-labeled LASP1 expression plasmid; HBV plasmid; the plasmids containing different HBV genes; cycloheximide (CHX); and antibodies against GAPDH, β-actin, HBsAg, HBX, p-AKT (Ser473), Flag-Tags, HA-Tags, and other reagents were obtained as previously reported (Tang et al., 2012; Kong et al., 2020; You et al., 2021; You et al., 2023a; You et al., 2023b). Antibodies targeting mTOR, COXIV, and p-mTOR were obtained from AbClonal (Wuhan, China). The antibodies against IL-32, SYVN1, AKT, PDK2, LASP1, and GLUD1 were purchased from Proteintech (Wuhan, China). PI3-K pathway activator SC79 (SF2730) and its inhibitor LY294002 (S1737) were obtained from Beyotime (Shanghai, China). The mTOR inhibitor (AZD8055) and PDK1 antibodies were purchased from Med-Chem Express (Shanghai, China). The plasmids containing shRNA against GLUD1 (sequence information: GCCATTGAGAAAGTCTTCAAA TTCAAGAGATTTGAAGACTTTCTCAATGGCTTTTTTG) and SYVN1 (sequence information: ACCGGTGCCAAGAGACTGCCCTGCAACTCGAGTTGCAGGGCAGTCTCTTGGTTTTTGA

ATTC), HA-labeled-GLUD1 expression plasmid, NEDD4L, STUB1, and SYVN1 expression plasmids containing Flag-Tags were purchased from Ke Lei Biological Technology (Shanghai, China). An shRNA plasmid targeting AKT (GATCCTCAAGAAGGAAGTCATTTCAAGAGAATGACTTCCTT CTTGAGGATCTTTTTT) was purchased from YouBio (Changsha, Hunan, China). Exogenous α-ketoglutarate (α-KG) (dimethyl-α-KG, a cell-permeable analog of α-KG) was purchased from Sigma Aldrich (St. Louis, MO, USA). The α-KG detection kit was purchased from Solarbio (Beijing, China). Proteins from the mitochondria and cytoplasm were extracted using a Mitochondria/Cytosol Protein Isolation Kit (Applygen Technologies, Beijing, China). HepG2 and Huh7 hepatoma cells were cultured, transfected with different plasmids, and selected using G418, as previously described (Kong et al., 2019; You et al., 2021).

Clinical samples, hematoxylin-eosin (HE) staining, and IHC analysis

HCC tissues (n=150), including 100 HBV-positive (HBV+) HCC tissues, 50 HBV-negative (HBV–) HCC tissues, and the associated adjacent normal tissues (n=60), were collected from Outdo Biotech Co., Ltd. (Shanghai, China). IHC was used to investigate the expression levels of different proteins in liver cancer tissues. Briefly, tissues were deparaffinized, rehydrated, and incubated with sodium citrate. Next, the tissues were treated with H_2O_2, blocked with goat serum, incubated with GLUD1 (1:200), LASP1 (1:200), SYVN1 (1:100), HBX (1:200) antibodies, and HRP-conjugated secondary antibodies (1:200), stained with 3,3'-diaminobenzidine, and terminated with water, Finally, the tissues were counterstained using hematoxylin. HE staining was performed as described previously (Yan et al., 2021). The expression levels of different proteins in the HCC tissues were determined using previously reported methods (Kong et al., 2020; Kong et al., 2021). This study was conducted in accordance with the principles of the Declaration of Helsinki. Approval was obtained from the Ethics Committees of Outdo Biotech Co., Ltd. and Xuzhou Medical University.

RNA sequencing and bioinformatics analysis

RNA sequencing was performed using the Illumina platform at Gene Denovo Biotechnology Co. (Guangzhou, China). The threshold for the differentially expressed genes was set based on the fold change at 2.0 and p=0.05. Gene expression information for GLUD1, as well as clinical data, including 50 normal tissues and 361 HCC tissues, were extracted from the TCGA database. The median was used as a cutoff value for classification into GLUD1 high and low expression groups. Additionally, the expression of GLUD1 and associated statistical data from different HCC cohorts were extracted from the HCCDB (Lian et al., 2018). Using Zhu cohort (Zhu et al., 2019), GLUD1 expression levels in the HBV-positive HCC tissues and HBV-negative HCC tissues were investigated. The GLUD1

gene expression information in HBV-positive HCC tissues and normal tissues was also extracted from Gao cohort (Gao et al., 2019). The interaction of LASP1 with other predicted proteins was collected from the UCSC genome browser gene interaction database, following the protocols provided on the database website (Mangan et al., 2014). Interactions between GLUD1 and other proteins were predicted using the PrePPI database (Zhang et al., 2013). The potential E3 ligases GLUD1 and LASP1 were predicted using the UbiBrowser database (version 1.0) (Li et al., 2017). The association between Kyoto Encyclopedia of Genes and Genomes (KEGG) pathway analysis and IL-32 expression was predicted using the ARCHS^4 database (Lachmann et al., 2018).

Animal transplantation

The target cells (2×10^7 cells/ml) were resuspended in phosphate-buffered saline (PBS). Matrigel solution (0.1 ml) combined with cell suspensions (0.1 ml) were injected into the null mice. The tumors in the mice were excised, and tumor weight and volume were assessed as reported previously (Kong et al., 2020; Kong et al., 2021). All experiments were approved by the Animal Care and Use Committee of Xuzhou Medical University.

Immunofluorescence analysis

The localization of GLUD1, AKT, LASP1, and SYVN1 in HCC cells was examined using immunofluorescence. Briefly, cells were fixed with ice-cold acetone, blocked using 2% bovine serum albumin (BSA) in PBS, incubated with GLUD1 (1:200), AKT (1:100), LASP1 (1:200), and SYVN1 (1:100) antibodies, washed, and incubated with secondary antibodies conjugated with Alexa Fluor 488 (1:200), Alexa Fluor 594 (1:100), and DAPI (1:200). Images were captured using an Olympus microscope.

Cell viability, cell clone formation, wound healing assays, and transwell experiment

Cell viability was examined using the CCK-8 Kit (Beyotime, Shanghai, China). Cell clone formation assay was performed with a 12-well culture plate (You et al., 2021). A wound healing experiment and transwell assay were performed, as previously reported (Tang et al., 2012; Kong et al., 2021).

Real-time polymerase chain reaction (PCR)

The primers used for GLUD1 were: GAAATCACCTACACCAGAGAGCC and TCATCCAGAAAAAATAAGCAAGC. PCR amplification conditions were: 4 min at 95 ℃, 30 cycles of 30 s at 95 ℃, 30 s at 60 ℃, as well as 30 s at 72 ℃. The expression of GLUD1 was normalized against that of GAPDH, the primers for which have been reported previously (Kong et al., 2015; Kong et al., 2020).

Western blot, Co‑IP, and ubiquitination assays

Western blotting, Co-IP, and ubiquitination experiments were performed as reported previously (You et al., 2021; You et al., 2023a). The cell extracts were incubated with GLUD1 (1:400), AKT (1:100), PDK1 (1:200), PDK2 (1:200), mTOR (1:500), LASP1 (1:500), SYVN1 (1:400) antibodies, and Protein G PLUS-Agarose (sc-2002, Santa Cruz Biotechnology, Santa Cruz, CA, USA). After the immunoprecipitates were washed, the target proteins were measured using western blotting. For the ubiquitination assay, extracts from target cells were incubated with Protein G PLUS-Agarose and ubiquitin antibodies (1:500, HuaBio, Hangzhou, China). Finally, western blotting was performed to examine the ubiquitination of the target proteins.

Statistical analysis

All values from at least three independent experiments are presented as mean ± standard deviation (SD), and the two-tailed unpaired t-test or one-way analysis of variance (ANOVA) test was performed to compare the differences. Chi-square test was used to determine the significance of the correlations between the target proteins detected by IHC. Univariate survival analysis with the log-rank test was performed to assess the association between disease-free survival (DFS), overall survival (OS), recurrence-free survival (RFS), and GLUD1 expression in HCC. Statistical significance was set at $P < 0.05$.

Supplementary References

Gao, Q., Zhu, H., Dong, L., et al. (2019). Integrated Proteogenomic Characterization of HBV-Related Hepatocellular Carcinoma. Cell 179, 561-577 e522.

Kong, F., Li, N., Tu, T., et al. (2020). Hepatitis B virus core protein promotes the expression of neuraminidase 1 to facilitate hepatocarcinogenesis. Lab. Invest. 100, 1602-1617.

Kong, F., Tao, Y., Yuan, D., et al. (2021). Hepatitis B Virus Core Protein Mediates the Upregulation of C5alpha Receptor 1 via NF-kappaB Pathway to Facilitate the Growth and Migration of Hepatoma Cells. Cancer Res. Treat. 53, 506-527.

Kong, F., You, H., Zhao, J., et al. (2015). The enhanced expression of death receptor 5 (DR5) mediated by HBV X protein through NF-kappaB pathway is associated with cell apoptosis induced by (TNF-alpha related apoptosis inducing ligand) TRAIL in hepatoma cells. Virol. J. 12, 192.

Kong, F., Zhou, K., Zhu, T., et al. (2019). Interleukin-34 mediated by hepatitis B virus X protein via CCAAT/enhancer-binding protein alpha contributes to the proliferation and migration of hepatoma cells. Cell Prolif 52, e12703.

Lachmann, A., Torre, D., Keenan, A.B., et al. (2018). Massive mining of publicly available RNA-seq data from human and mouse. Nat. Commun. 9, 1366.

Li, Y., Xie, P., Lu, L., et al. (2017). An integrated bioinformatics platform for investigating the human E3 ubiquitin ligase-substrate interaction network. Nat. Commun. 8, 347.

Lian, Q., Wang, S., Zhang, G., et al. (2018). HCCDB: A Database of Hepatocellular Carcinoma Expression Atlas.

Genomics Proteomics Bioinformatics 16, 269-275.

Mangan, M.E., Williams, J.M., Kuhn, R.M., et al. (2014). The UCSC Genome Browser: What Every Molecular Biologist Should Know. Curr. Protoc. Mol. Biol. 107, 19.19.11-19.19.36.

Tang, R., Kong, F., Hu, L., et al. (2012). Role of hepatitis B virus X protein in regulating LIM and SH3 protein 1 (LASP-1) expression to mediate proliferation and migration of hepatoma cells. Virol. J. 9, 163.

Yan, C., Zhou, Q.Y., Wu, J., et al. (2021). Csi-let-7a-5p delivered by extracellular vesicles from a liver fluke activates M1-like macrophages and exacerbates biliary injuries. Proc. Natl. Acad. Sci. U. S. A. 118, e2102206118.

You, H., Yuan, D., Bi, Y., et al. (2021). Hepatitis B virus X protein promotes vimentin expression via LIM and SH3 domain protein 1 to facilitate epithelial-mesenchymal transition and hepatocarcinogenesis. Cell Commun. Signal 19, 33.

You, H., Yuan, D., Li, Q., et al. (2023a). Hepatitis B virus X protein increases LASP1 SUMOylation to stabilize HER2 and facilitate hepatocarcinogenesis. Int. J. Biol. Macromol. 226, 996-1009.

You, H., Zhang, N., Yu, T., et al. (2023b). Hepatitis B virus X protein promotes MAN1B1 expression by enhancing stability of GRP78 via TRIM25 to facilitate hepatocarcinogenesis. Br. J. Cancer 128, 992-1004.

Zhang, Q.C., Petrey, D., Garzon, J.I., et al. (2013). PrePPI: a structure-informed database of protein-protein interactions. Nucleic Acids Res. 41, D828-833.

Zhu, Y., Zhu, J., Lu, C., et al. (2019). Identification of Protein Abundance Changes in Hepatocellular Carcinoma Tissues Using PCT-SWATH. Proteomics. Clin. Appl. 13, e1700179.

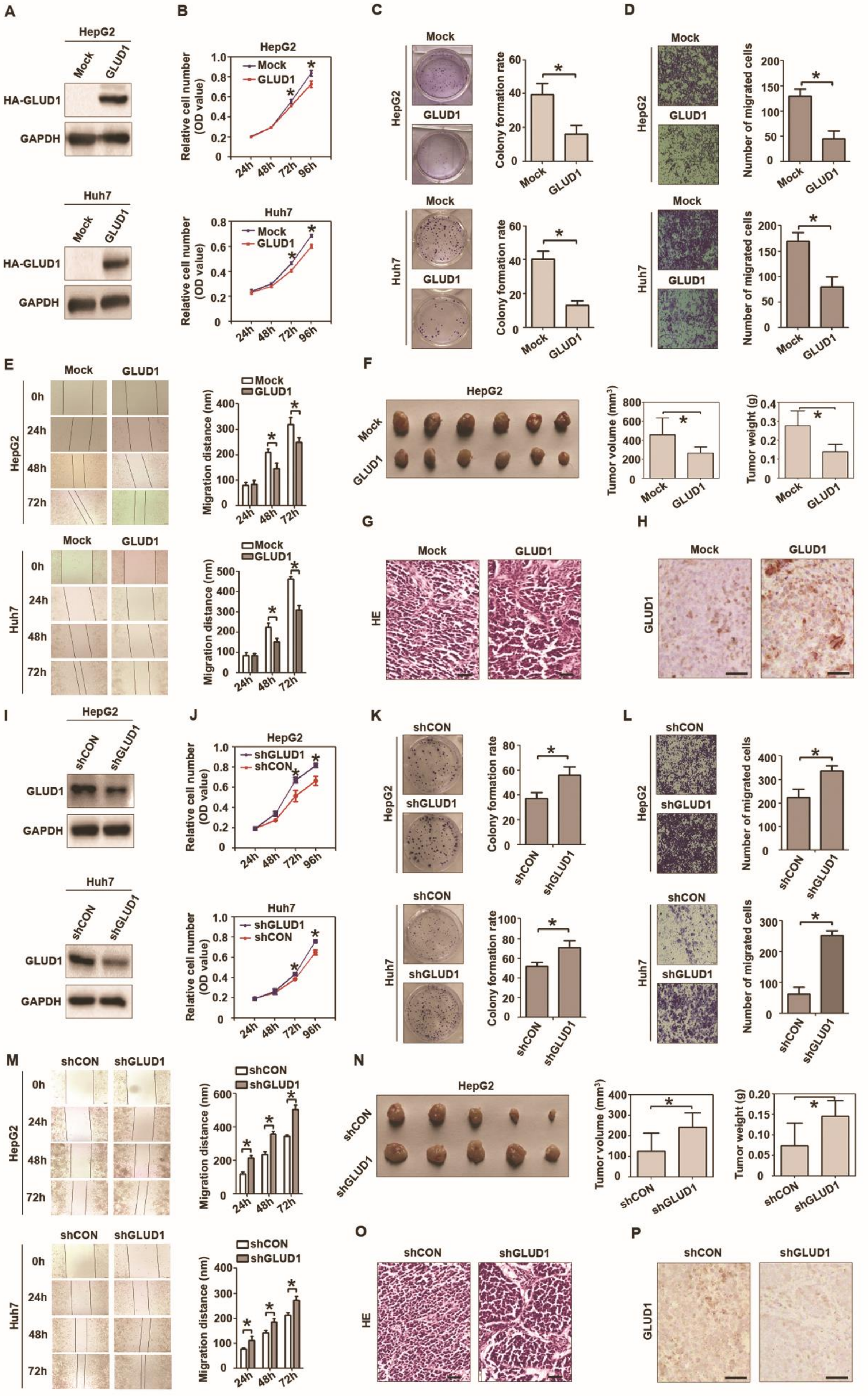
A
HepG2
Mock
GLUD1
HA-GLUD1
GAPDH
Huh7
B
HepG2
Relative cell number (OD value)
Mock
GLUD1
24h
48h
72h
96h
Huh7
C
Mock
GLUD1
HepG2
Huh7
Colony formation rate
D
Number of migrated cells
E
0h
24h
48h
72h
Migration distance (nm)
F
Tumor volume (mm3)
Tumor weight (g)
G
HE
H
I
shCON
shGLUD1
GLUD1
J
K
L
M
N
O
P

Supplementary Figure S1 The effect of GLUD1 on the proliferation and migration of hepatoma cells. (**A**) Exogenous expression of HA-labeled GLUD1 in HCC cells. (**B**) CCK-8 assay showed the role of GLUD1 overexpression in HCC cell proliferation. (**C**) Cell cloning experiment showed the role of GLUD1 overexpression in HCC cell proliferation. (**D**) Transwell assay showed the effect of GLUD1 overexpression in HCC cell migration. (**E**) Wound healing experiment showed the effect of GLUD1 overexpression in HCC cell migration. (**F**) The effect of GLUD1 overexpression in the growth of HCC in nude mice. (**G**) HE staining of xenograft tumor tissues from GLUD1 overexpression group and its control group. Scale bar: 50 μm. (**H**) The expression of GLUD1 was detected by IHC in xenograft tumor tissues from the GLUD1 overexpression group and its control group. Scale bar: 50 μm. (**I**) The inhibition of GLUD1 expression by its specific shRNA in HCC cells. (**J**) CCK-8 assay showed the role of GLUD1 silencing in HCC cell proliferation. (**K**) Cell cloning experiment showed the role of GLUD1 silencing in HCC cell proliferation. (**L**) Transwell assay showed the effect of GLUD1 silencing in HCC cell migration. (**M**) Wound healing experiment showed the effect of GLUD1 silencing in HCC cell migration. (**N**) The effect of GLUD1 silencing in the growth of HCC in nude mice. (**O**) HE staining of xenograft tumor tissues from GLUD1 silencing group and its control group. Scale bar: 50 μm. (**P**) The expression of GLUD1 was detected by IHC in xenograft tumor tissues from the GLUD1 silencing group and its control group. Scale bar: 50 μm. Mock: the HCC cells transfected with control expression plasmids, GLUD1: the HCC cells transfected with HA-labeled GLUD1 expression plasmids, shCON: the HCC cells transfected with shRNA control plasmids, shGLUD1: the HCC cells transfected with shRNA targeting GLUD1 plasmids, $^{*}P < 0.05$

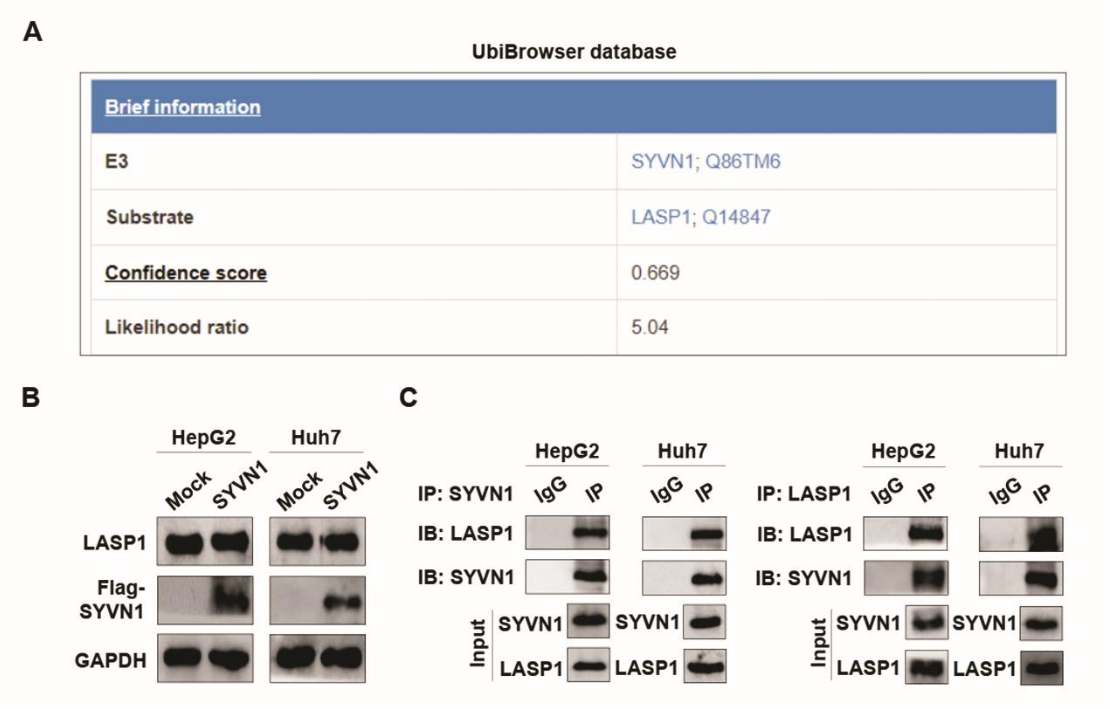

Supplementary Figure S2 The role of SYVN1 on LASP1 expression and the interaction of SYVN1 with LASP1. (**A**) SYVN1 acts as a predicted E3 ligase of LASP1, based on the UbiBrowser database. (**B**) The effect of exogenous SYVN1 on LASP1 expression in HCC cells. (**C**) The interaction between SYVN1 and LASP1 detected by Co-IP assay.

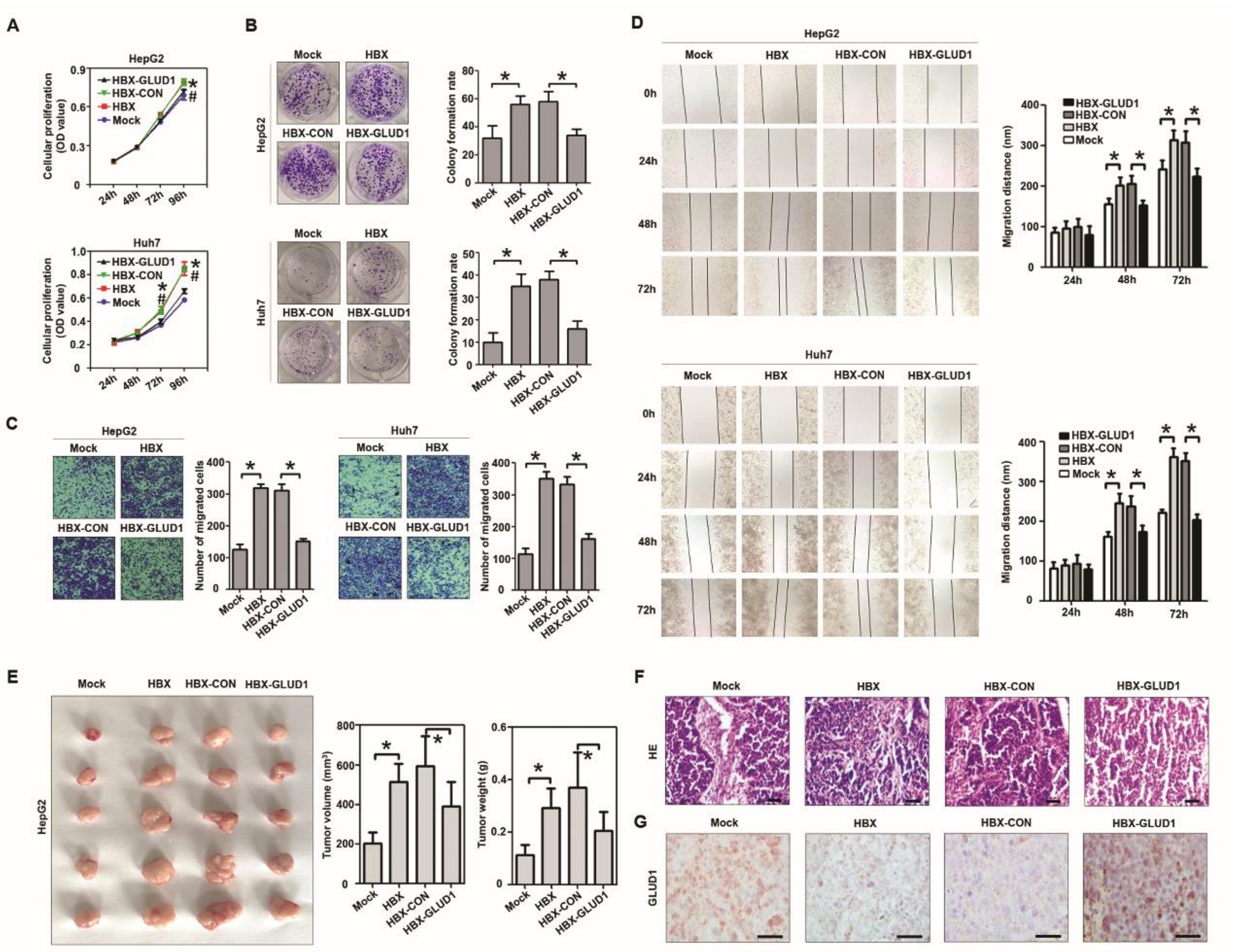

Supplementary Figure S3 The effect of GLUD1 on the proliferation and migration of HBX-positive hepatoma cells. (**A**) CCK-8 assay showed the role of GLUD1 in HCC cell proliferation induced by HBX. (**B**) Cell cloning experiment showed the role of GLUD1 in HCC cell proliferation induced by HBX. (**C**) Transwell assay showed the effect of GLUD1 in HCC cell migration induced by HBX. (**D**) Wound healing experiment showed the effect of GLUD1 in HCC cell migration induced by HBX. (**E**) The effect of GLUD1 in the growth of HBX-positive HCC in nude mice. (**F**) HE staining of HBX-positive xenograft tumor tissues and associated tumor tissues. Scale bar: 50 µm. (**G**) The expression of GLUD1 detected by IHC in HBX-positive xenograft tumor tissues and associated tumor tissues. Scale bar: 50 µm. Mock: the HCC cells transfected with control expression plasmids, HBX: the HCC cells transfected with HBX expression plasmids, HBX-CON, the HBX-positive HCC cells transfected with control expression plasmids, HBX-GLUD1, the HBX-positive HCC cells transfected with GLUD1 expression plasmids, *$P < 0.05$, HBX group compared to Mock group, #$P < 0.05$, HBX-GLUD1 group compared to HBX-CON group.

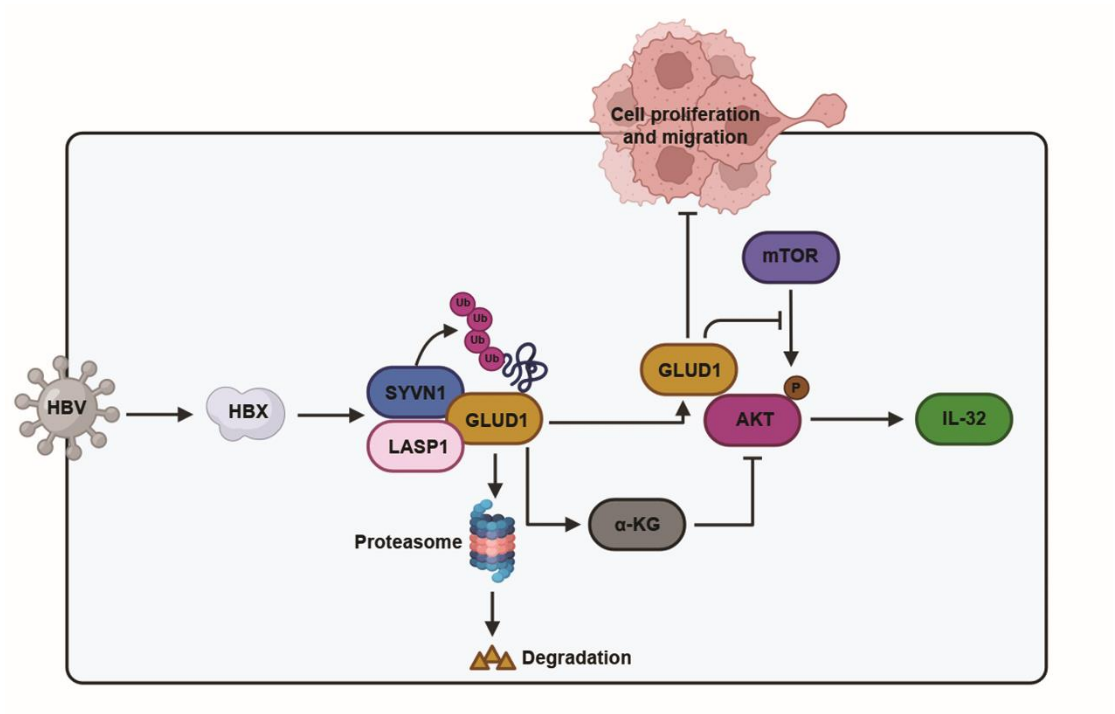

Supplementary Figure S4 A schematic diagram showing the mechanisms associated with the down-regulation of GLUD1 induced by LASP1 and SYVN1 to activate AKT and upregulate IL-32 to facilitate HBX-associated hepatocarcinogenesis. The schematic diagram was created with BioRender.com (https://biorender.com/).
